# New polyvinyl alcohol/gellan gum-based bioplastics with guava and chickpea extracts for food packaging

**DOI:** 10.1038/s41598-023-49756-0

**Published:** 2023-12-16

**Authors:** Shaimaa Elsaeed, Elsayed Zaki, Ayman Diab, Menna-Alla Tarek, Walaa A. E. Omar

**Affiliations:** 1 Egyptian Petroleum Research Institute, Naser City, Cairo, 11727 Egypt; 2 Faculty of Biotechnology, October University for Modern Sciences and Arts, 6th of October City, Egypt; 3 Faculty of Petroleum and Mining Engineering, Suez University, P.O.Box: 43221, Suez, Egypt

**Keywords:** Climate sciences, Environmental sciences, Chemistry, Materials science

## Abstract

Plastic is a fossil-based synthetic polymer that has become an essential material in our daily life. Plastic pollution resulting from the accumulation of plastic objects has become problematic for our environment. Bioplastic can be a biodegradable environmentally friendly alternative for the synthetic plastic. In this paper, bioplastics based on polyvinyl alcohol (PVA)/gellan gum (GG) blend have been produced in three different compositions and their chemical structure, mechanical, morphological and thermal properties have been studied. Glycerol has been used as a plasticizer. To add extra features to the PVA/GG bioplastic, Psidium guajava (guava) leaves, GL, and chickpea, CP, extracts have been added to the PVA/GG (30/70) blend. Water and aqueous ethanol have been used in the extraction of GL and CP, respectively. The addition of the plant’s extracts enhanced the tensile properties of the PVA/GG bioplastic. Weathering acceleration tests have been carried out to examine the degradation of the prepared bioplastics. Cytotoxicity studies revealed that the prepared bioplastic is safe to be used in food packaging applications. Water and oxygen permeability for the new PVA/GG bioplastic have also been studied. The addition of the plant extracts (GL and CP extracts) increased the oxygen and water permeability to different extents. Bioplastic life cycle assessment (LCA) and CO_2_ emissions in comparison to fossil-based plastic have been investigated. From all the results, PVA/GG based bioplastic proved to be a degradable, safe and effective alternative for fossil-based plastics in food packaging applications.

## Introduction

Plastics are petroleum based organic polymers that possess extraordinarily rapid growth and large-scale production due to their lightweight, chemical stability and low processing cost^1–3^. This massive production is derived by the global shift from reusable to single use containers. On the other hand, the huge plastic production generated a global solid waste accumulation that is growing steadily over the years^4^. There are two basic classes of plastics, thermoplastics and thermosets. For both classes of plastics, most of the monomers used to produce them are derived from fossil hydrocarbons such as ethylene, propylene, and styrene. Therefore, plastics are non-biodegradable i.e., they don’t decompose naturally. Consequently, plastic accumulates in the earth and oceans causing serious environmental and health problems^1–5^ .

**Figure 1:**
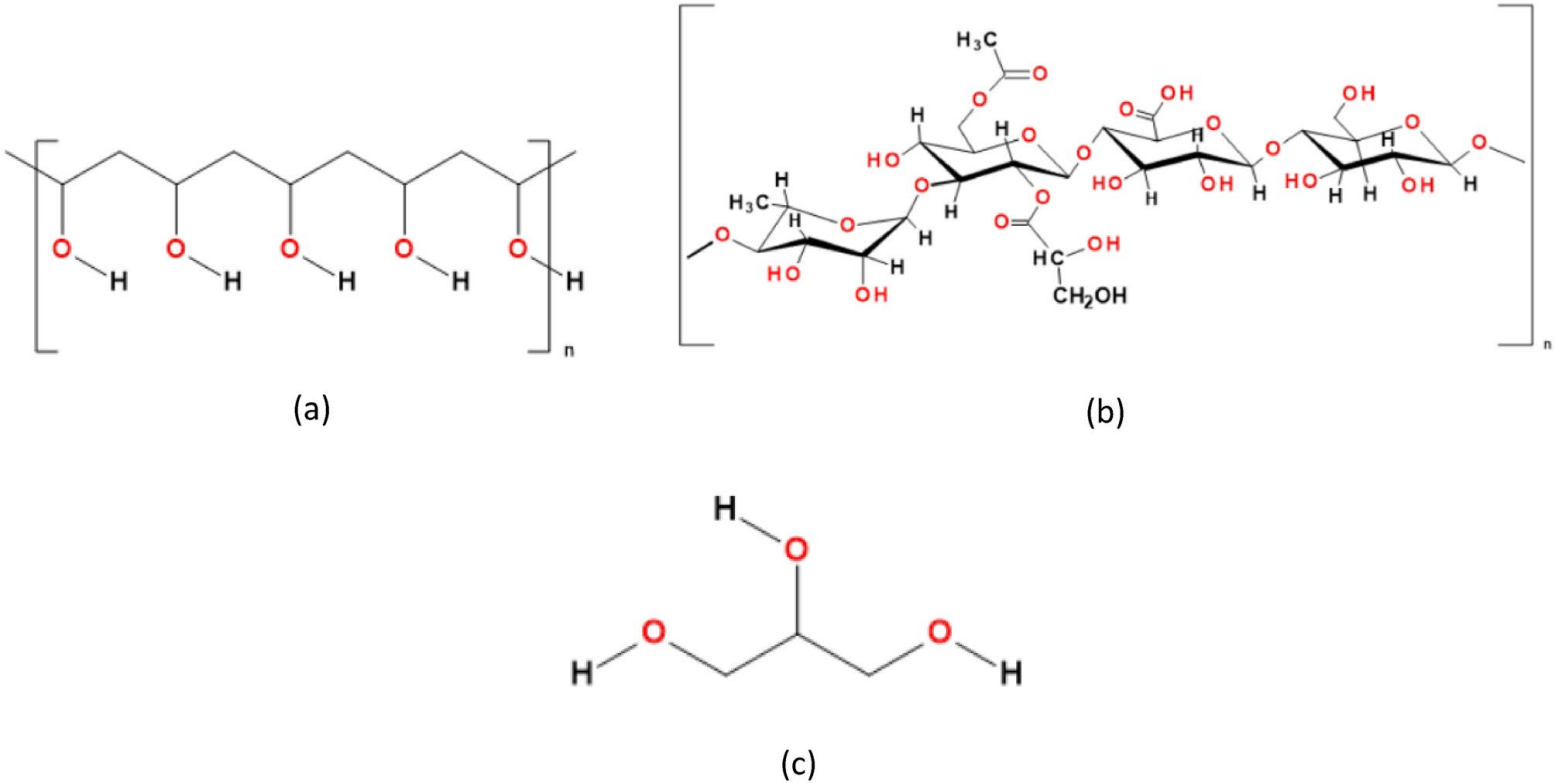
(**a**) The chemical structure of PVA^1^, (**b**) GG^20,21^, and (**c**) Glycerol.

In contrast to plastics, bioplastics includes polymers that are biodegradable and/or originate from renewable resources^2,6^. They can be classified into four classes, Class (I) which includes the polysaccharides that are directly extracted from biomass such as starch^7,8^, xanthan gum^9^, guar gum^10^, gellan gum and proteins. Class (II) of bioplastics includes materials that are bio-sourced but not biodegradable such as polyethylene produced from ethanol by fermentation^2^. Class (III) of bioplastics includes materials that are produced from petrochemicals but are degradable. An example of such materials is polyvinyl alcohol (PVA, the structure is illustrated in Fig. 1a). Class (IV) are polymers that are produced by the action of bacteria such as polyhydroxyalkanoate^2^.

Bioplastics made from blends of two or more types of the above-mentioned polymers have been a target of many research groups aiming to have enhanced/desired mechanical properties and/or lower cost^2,11,12^.

Polyvinyl alcohol (PVA, Fig. 1a)^1^, which belongs to class (III) in bioplastics classification^2^, is a water-soluble synthetic biodegradable polymer. It is widely used as thermoplastic polymer and is usually prepared by hydrolysis of polyvinyl acetate. PVA is nontoxic and has many applications in industry, medicine, and food sector, specifically food packaging^13^. Physical mixing of synthetic polymers such as PVA with natural polymers represents an effective way to obtain polymeric materials with desired features, improved mechanical characteristics and biological performances^14^. For that purpose, PVA was blended successfully with many natural polymers and the produced copolymers are successfully employed in many fields. In medicine, PVA blends were promising candidates in tissue engineering. PVA/chitosan blend has been employed in neural tissues therapies^15^. While PVA/PET (polyethylene terephthalate) blend has been applied in vascular grafts, PVA/collagen has been used in corneal applications. PVA/Gellan gum (GG) biodegradable nanofibers have been prepared for skin tissue regeneration^11,12^.

In the same context, films formed from such PVA blends showed enhanced mechanical properties for industrial applications^16^. The films mechanical properties could be tuned by the PVA percentage in the blend. PVA blends with natural polymers such as guar gum^17^, starch^1,18^, cellulose^19^ and chitosan^20^ have been reported in food packaging applications. PVA is soluble in water due to the presence of the hydroxyl groups in its backbone (Fig. 1a)^1^. The PVA hydrophilicity enables the use of water as a solvent during blending PVA with other polymers. The water temperature should be around 100 °C to have the PVA completely soluble and ready to be mixed with other dissolved polymers forming a homogenous solution^16^.

Brunchi et al*.*^14^ studied the interaction in the blend PVA /xanthan gum. FTIR analysis proved the formation of a network of hydrogen bonds between the carboxylate and hydroxyl groups in xanthan gum and the hydroxyl groups of PVA.

Gellan gum (GG) is a biocompatible thermally stable polysaccharide (Fig. 1b). It is a hydrophilic biopolymer that is widely recognized as a food additive^21,22^. However, it has poor mechanical strength, high gelling temperature and small temperature window. Therefore, GG was blended with different natural and synthetic polymers to have a blend with tunable mechanical and physical properties^11,12^.

Paolicelli et al.^23^ have prepared thin films of plasticized GG by solvent casting technique for oral delivery applications. They reported that the use of glycerol (Fig. 1c) as a plasticizer by an amount of 20–75% w/w enhanced the mechanical characteristics of GG based films as it reduced the interconnection among the polymeric chains. In general, glycerol is a biodegradable, non-toxic trihydroxy compound that is widely used as a plasticizer^24,25^.

PVA\GG composites have been the target of a few studies. Vashisth et al.^12^ prepared electro-spun PVA\Gellan nanofibers. The composite has been employed in biomedical engineering after stabilization by cross-linking under different conditions.

Sudhamani et al.^26^ reported PVA/GG blended films by solution casting method. They used water as solvent and glycerol as a plasticizer. The thermal characteristics, intermolecular interactions and mechanical properties have been studied by differential scanning calorimetry (DSC), Fourier transfer spectroscopy (FTIR) and tensile tests, respectively. Although the mechanical properties suggested that films from that blend are potential candidates for food packaging, no extended real performance or hydro-stability tests were provided.

Bioplastic with extra features and properties that suit the packaging purpose would be highly desirable. Research groups have incorporated plants extract to add value to bioplastics. Tran et al*.*^27^ added oregano waste extract to propylene carbonate/cellulose acetate bioplastic to obtain high UV protection and antioxidant food packaging. Jung et al.^28^ mixed a naturally produced indigo pigment with polyhydroxyalkanoate to produce blue colored bioplastic. Pea protein-based bioplastics have also been produced. This type of bioplastics possessed enhanced mechanical and antimicrobial properties^29^.

Among the plants, *Psidium guajava* (Guava) leaves extract, which is denoted as GL in this paper, has several biological activities. It has antifungal, antimicrobial, anti-cancer, antioxidant, and antitumor properties^30–32^. Moreover, the bioactive compounds extracted from guava leaf have been used in the preparation of jelly referred as guava leaf extract jelly. Adequate intake of these jellies helped the consumer lower high cholesterol levels in the body and boost up immunity^33^. Guava leaves extract have been recently added to sodium alginate to develop antimicrobial food packaging^34^.

Chickpeas (*Cicer arietinum*), CP, have got several applications in food, cosmetics, and packaging^35^, in addition to their applications in medicine such as the treatment of ulcer, bronchitis, cholera, constipation, and cancer^36,37^. Chickpea aquafaba has also been recently employed in bioplastic packaging^35^. Additionally, the Proteins isolated from the Chickpea seeds were good candidates for thermoplastic biopolymeric materials^38^.

Bioplastic for food packaging might be ingested by human and animals. Additionally, most food packages are designed for single use and very probably will be disposed to the environment. Therefore, materials to be used for food packaging applications should be safe to consumers and the environment^39–41^.

In this paper, PVA\GG composites have been prepared with three different compositions (50/50, 30/70, and 70/30) which are denoted in this research as S1, S2 and S3, respectively. Films from the blends have been prepared by solvent casting technique. The bioplastic films were characterized by FTIR, and the mechanical properties were investigated by tensile tests. Plant extracts, namely, Guajava leaves (GL), and chickpeas (CP) extracts have been prepared and characterized by Fourier transform infrared spectroscopy. The extracts were incorporated individually to the most promising composition of the PVA/GG blends (S2 composition). Films of PVA/GG and the plant extracts (S2/GL and S2/CP) have been investigated for their food packaging properties and utilization. To test the real performance of the prepared films as food packaging material, accelerated weather testing and water wettability tests have been carried out. The bioplastic films have also been tested for their cytotoxicity, oxygen, and water permeability. Life cycle assessment (LCA) and CO_2_ emissions of the newly prepared bioplastics have been studied.

## Methodology

### Chemicals and plant materials

#### Chemicals

PVA and GG were purchased from Sigma-Aldrich, USA. Glycerol was purchased from the united company for chemicals, Cairo.

#### Plant materials

The plant materials collection and use in this research comply with the national and international guidelines and legislation. GL were collected from national Guajava trees in Menofia. Chickpeas were commercial grade and were purchased from national food store.

### Bioplastic films preparation

#### PVA/GG bioplastic films preparation

PVA/GG films have been prepared by solvent casting technique^25^. PVA solution was prepared by dissolving 5 g of PVA in 5 mL of distilled water and heated at 90 °C with stirring. GG, 2 g, was dissolved in 2 mL distilled water and stirred at 70 °C until a clear solution was obtained. The two solutions were then mixed and stirred for 3–4 h to obtain a completely homogeneous solution. Glycerol, 2 mL, was then added to the blend and stirred at room temperature for 1 h. The mixture was then poured onto a clean and dry petri dish (200 × 20 mm) and dried in an oven at 50 °C. This film with PVA/GG composition 70/30 is denoted as S3 in Table 1. The same procedures have been repeated to prepare PVA/GG films with compositions 30/70 and 50/50 which were denoted as S2 and S1, respectively. After drying, the films were peeled off and stored in a desiccator for further characterization.

**Table 1:** The different compositions of GG/PVA blends.

Sample	PVA (wt%)	GG (wt%)	Glycerol (mL)
S1	50	50	2
S2	30	70	2
S3	70	30	2

#### PVA/GG/GL film preparation

Hot water (100 mL) at 85 °C has been added to 2 g of dry powdered guajava leaves and left to soak for 24 h. The extract was then filtered and added to the PVA/GG blend of composition 2 (S2) in 5 wt% and stirred for ½ hr. The blend PVA/GG/GL was poured onto clean and dry petri dish (200 × 20 mm) and dried in an oven at 50 °C for 2 days. The film was then peeled off and stored in a desiccator for further characterization.

#### PVA/GG/CP film preparation

Ethanol–water mixture (1:1, 100 mL) was added to 2 g of finely powdered chickpea seeds and soaked for 24 h. The extract was then filtered and added to the PVA/GG blend with composition 2 (S2) in 5 wt%. The PVA/GG/CP blend was stirred for ½ hr, poured onto clean and dry petri dish (200 × 20) and dried in an oven at 50 °C for 2 days. The film was then peeled off and stored in a desiccator for further characterization.

### Fourier transform infrared spectroscopy (FTIR)

The FTIR of all the films were recorded on a nicolet iS10 FT-IR spectrophotometer with a resolution of 4 cm^-1^ in the range 450 and 4000 cm^-1^.

### Mechanical measurements

Tensile tests were performed by the ASTM D412 test strategy and were done at room temperature using a tensile tester (Tinius Olsen-H10K). The crosshead speed was (10 mm/min). The initial gauge length of the specimen was (80 mm) and the width of each sample was (2 mm). Thickness 0.130–0.150 mm.

### Contact angle measurement

The contact angle measurements were performed using Attention Theta Optical Tensiometer (Biolin Scientific Company, Finland).

### Cytotoxicity studies for PVA/GG film, S2

Determination of S2 (PVA/GG, 30/70 wt%) cytotoxicity on Vero cells (kidney tissue) using MTT protocol has been carried out following the procedures reported elsewhere^42^ at a concentration 1000 ug/mL of S2.

### Scanning electron microscopy (SEM) for the PVA/GG (S2) film

The surface morphology of the prepared PVA/GG film of composition 30/70 wt% (S2) was examined using FEI Quanta FEG 250 high resolution scanning electron microscope (SEM).

### Water vapor transmission rate (WVTR) measurements

The WVTR measurements for S2, S2/GL and S2/CP films were performed according to the method reported in^43^ using a samples of 1.5 cm diameter. The films under investigation were sealed on top of a glass bottle filled with distilled water and placed in a desiccator at 90% relative humidity. The decrease in the weight was detected using a microbalance at 24 h time intervals for 5 days. All experiments were carried out in duplicate. The WVTR values were calculated from the slope of the regression line of the weight change/time graph using Eq. (1)^43^.

1 $$WVTR = \Delta \, m/\left( {A \times t} \right)$$

Where *(Δ m/t)* is the slope of the weight change against time (g/day), *A* is the sample exposed surface area in m^2^.

### Oxygen permeability

The oxygen permeabilities of samples S2, S2/GL and S2/CP were measured by the method described by Shi et al.^44^ The oxygen permeability of the samples under investigation has been calculated using Eq. (2)^44^.

2 $$OP\left( {oxygen \, \;permeability} \right) = \left( {M_{f} {-} \, M_{i} } \right)/(t \, x \, A)$$

where *M*_*f*_ is the final weight of the weighing bottle after equilibrated in the desiccator for 24 h at 90% humidity and 25 °C, *M*_*i*_ is the initial weight of the weighing bottle and ***t*** is the equilibrated time (48 h) in seconds, and *A* is the surface area of the exposed films.

#### Accelerated weather testing

The accelerated weathering of the bioplastic samples was conducted in an accelerated weathering tester Model QUV/se (Q-LAB, Westlake, Ohio, USA). The weathering conditions were following the Cycle-C of the ASTM D6164 standard. Fluorescent UV lamps (UV-A-340) with 0.76 W m-2 irradiance (wavelength 340 nm) were used with cycles of 8 h UV irradiation at 50 °C, followed by 4 h dark at 50 °C under 100% condensing humidity. These consecutive cycles were applied to the specimens attached to the test panels without any interruption. The effects of accelerated weathering were investigated for 100 h exposure periods.

#### Life cycle assessment and carbon footprint for the prepared bioplastic

Life cycle assessment has been investigated using the model proposed by Benavides et al.^45^. The data for CO_2_ emissions for the different components in the bioplastic have been collected from different resources. The references are provided in the references section for each value considered.

## Results and discussion

### preparation of PVA-GG blend

PVA/GG blends were prepared by blending PVA (Fig. 1a) and GG (Fig. 1b) in three different compositions, (50/50), (30/70) and (70/30) wt% denoted as S1, S2 and S3, respectively, Table 1.

**Figure 2:**
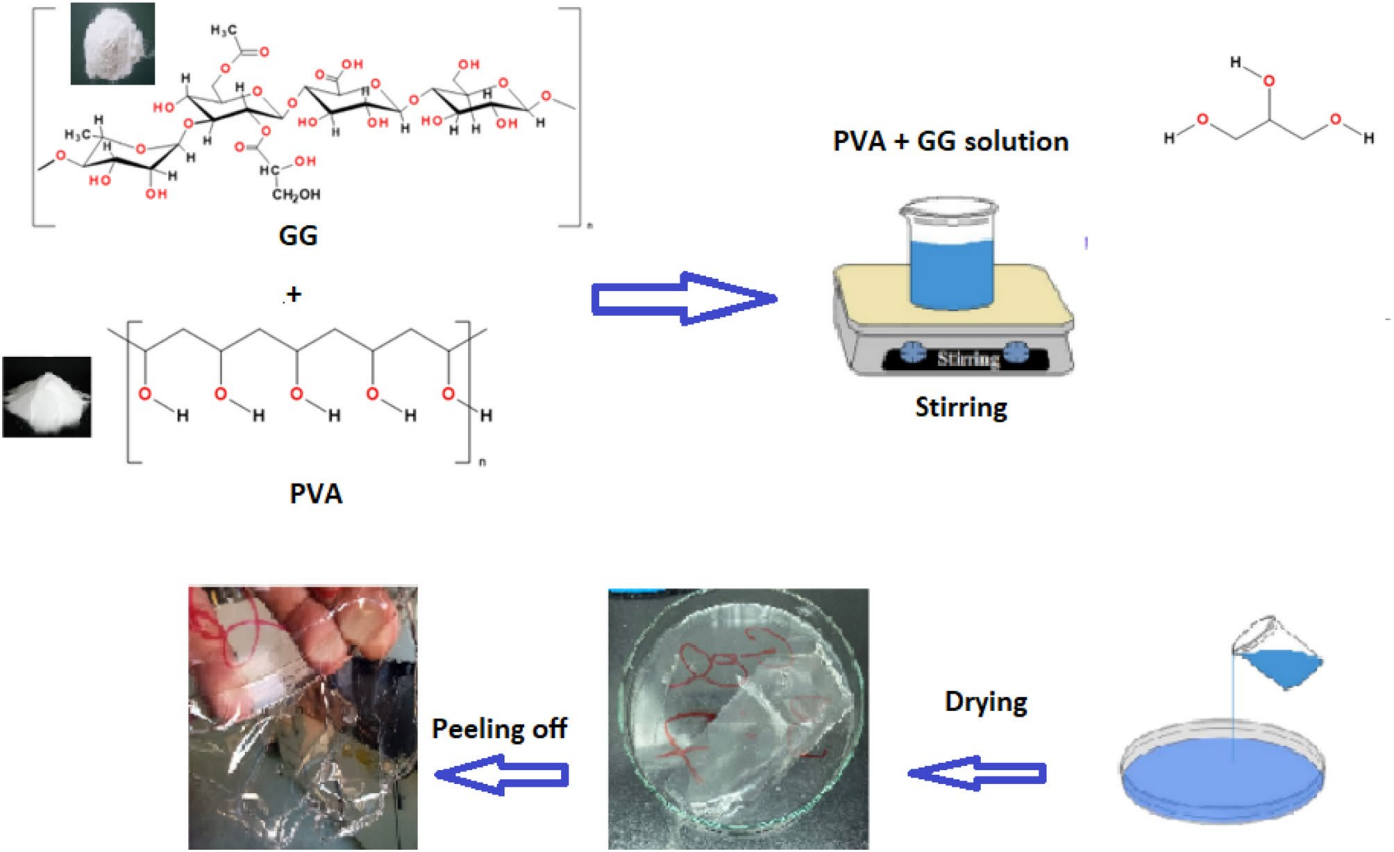
The step wise preparation of GG/PVA bioplastic by solvent casting techniques.

It has been reported that, addition of glycerol by 20–70% to GG based bioplastic films enhanced the mechanical properties^23^, therefore, glycerol, 25 wt%, was added to the three compositions as a plasticizer. Solvent casting technique^26^ has been used to prepare the bioplastic films, where the blends have been poured uniformly onto dry and clean petri dishes and allowed to dry in an oven. After peeling, the films of the different compositions have been kept in a desiccator for further characterization (Fig. 2).

The films of PVA/GG with the three compositions (S1, S2 and S3) have been characterized by FTIR. The mechanical properties have also been investigated by tensile strength measurements.

### Fourier transform infrared spectroscopy (FTIR) for the PVA/GG blends

**Figure 3:**
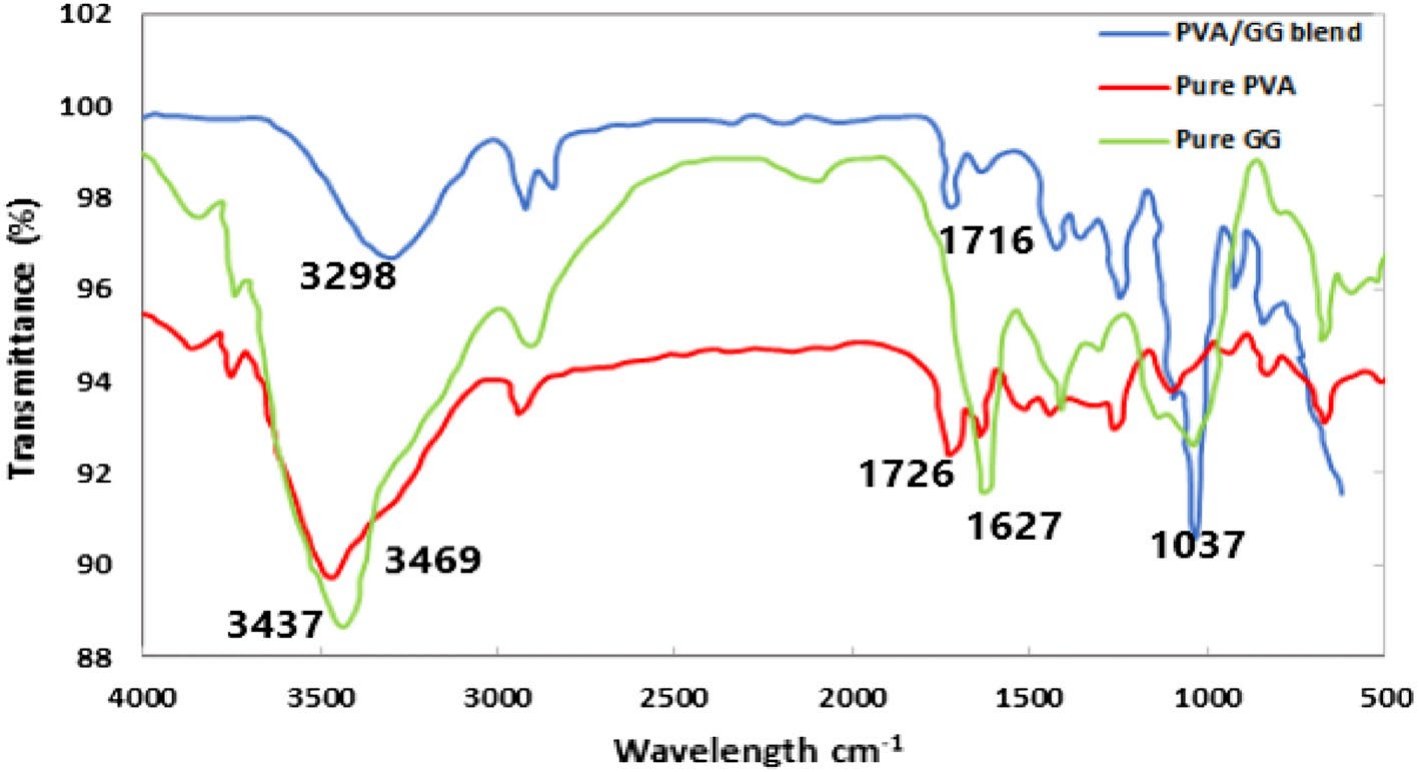
The FTIR for pure GG (green line), Pure PVA (red line) and PVA/GG blend (blue line).

The FTIR for the pure GG and PVA, in addition to the three blends have been investigated. The three compositions of PVA/GG (S1, S2 and S3) gave almost the same bands in the IR spectra; therefore, the three compositions have been represented by only one IR chart in Fig. 3. Figure 3 shows the IR for the pure GG, PVA and GG/PVA blend. FTIR chart for pure GG showed the characteristics bands for GG. The large broad band at 3437 cm^-1^ is corresponding to the -OH group vibration and the stretching of intermolecular hydrogen bonding. At 2891 cm^-1^, is the CH_3_ and CH_2_ stretching vibrations. The band at 1627 cm^-1^ is probably corresponding to the C=O stretching while the band at 1031 cm^-1^ represents the glycosidic bond vibration. The band at 1412 cm^-1^ corresponds to the CH bending^46,47^. The FTIR chart of the pure PVA is also shown in Fig. 3. The peak at 3469 cm^-1^ represents the -OH group asymmetric stretching vibration resulting from the inter- and intramolecular hydrogen bonding in pure PVA. The peak at 2941 cm^-1^ refers to the –CH stretching from the alkyl groups. The peak at 1726 cm^-1^ corresponds to the –C=O stretching. The peak at 1450 cm^-1^ represents –CH bending while at 1100 cm^-1^ is due to the -CO stretching of the –COH group^48–50^. After blending GG and PVA, a shift in the peak positions was observed. The peak assigned to the -OH was broader and shifted to a lower wavelength (3298 cm^-1^) than in the pure PVA and GG. This shift can be attributed to an increase in the intermolecular hydrogen bonding resulted from the blending. The peak at 1726 cm^-1^ in PVA was also shifted to be at 1716 cm^-1^ which indicates an increase in the hydrogen bonding. The peak at 1031 cm^-1^ in GG appeared at 1037 cm^-1^ in the mixture which indicates successful blending^23,49^.

### Guava extraction, characterization, and incorporation in the PVA/GG blend

It has been reported that Guava trees contribute largely to the CO_2_ sequestration. They help in the reduction of greenhouse gas emissions in the atmosphere^51^. A 10–15 years old Guava tree can remove from 12 to 13 kg/year of CO_2_ from the atmosphere^52^. Therefore, in this work, we incorporated GL extract to the PVA/GG bioplastic in order to reach a reduced net GHG emissions and consequently reduce the CO_2_ footprint of the prepared bioplastic. Additionally, the GL extract would enhance the mechanical and the bioactive properties of the bioplastic for food packaging applications^30–34^.

**Figure 4:**
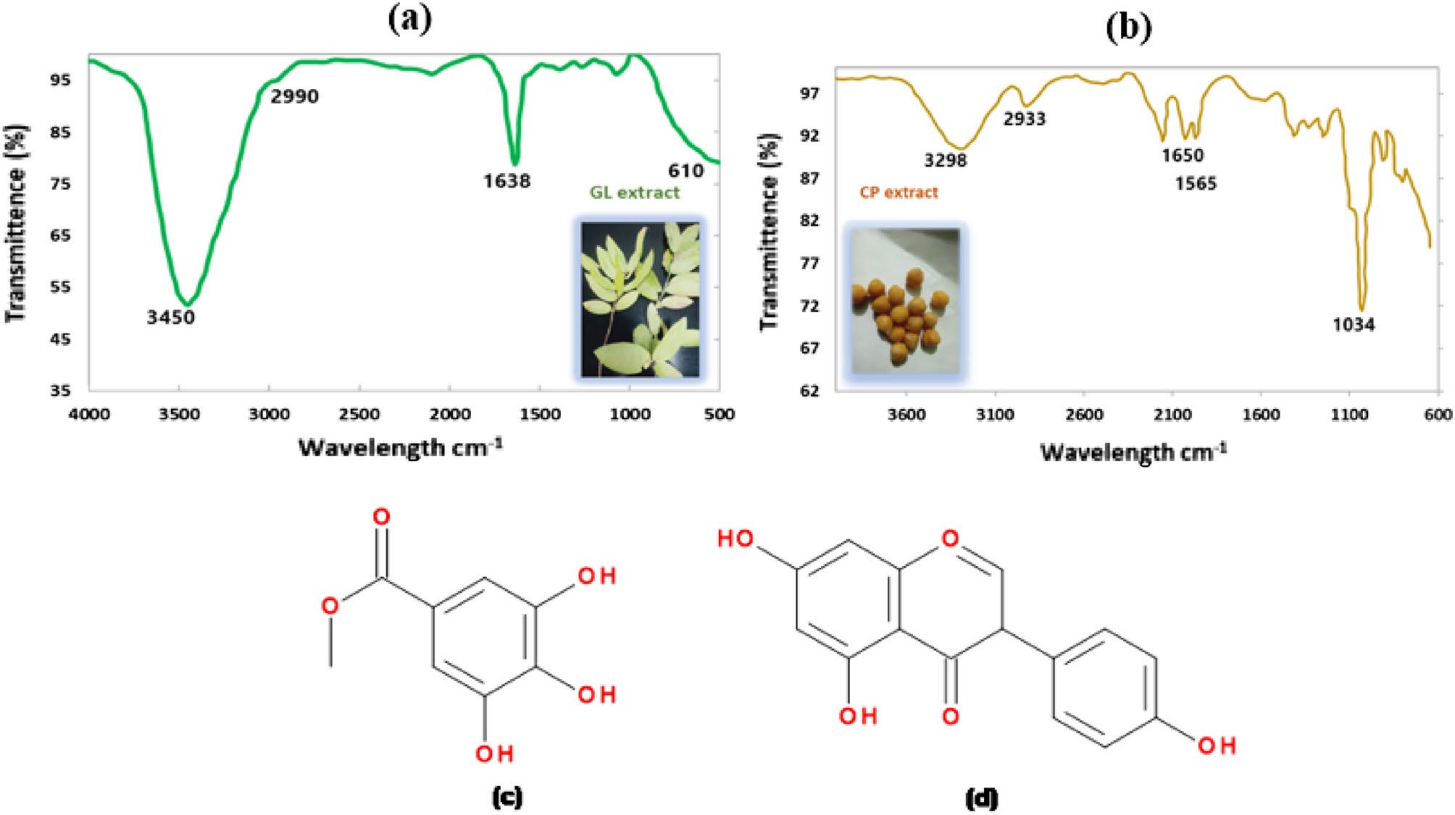
(**a**) the FTIR of GL extract, (**b**) FTIR of CP extract, (**c**) the galloyl group present in GL extract active compounds^50^, and (**d**) an example of the hydroxy compounds presents in CP extract (genistein)^61^.

As the type of solvent used in plants extraction affects the amount of the bioactive material in the extract. Water was the most commonly used solvent for extraction of the active compounds from GL because it is safe and economic^53^. Therefore, in this paper, GL were extracted in hot distilled water. The leaves were dried before extraction. This is due to the fact that the phenolic compounds and antioxidants are more concentrated in dried plants than in fresh ones^53,54^. It has been reported that water extract of GL is rich in glycosides, saponins, tannins, phenols, terpenoids and flavonoids^31,33^. The aqueous GL extract has been characterized by FTIR, Fig. 4a. The broad band at 3450 cm^-1^ refers to the -OH stretching of the phenolic compounds. The band at 2990 cm^-1^ is attributed to the -CH stretching of aliphatic hydrocarbons. At 1638 cm^-1^ is the –C=O stretching of the carbonyl compounds. A band at 1070 cm^-1^ corresponds to the –CO of the flavonoids. The broad band at 610 cm^-1^ corresponds to the heterocyclic compounds in GL extract^31,53^.

In GL water extract, the –OH and .C=O functionalized compounds are predominant. The chemical structure of the most abundant group present in GL extracts compounds is illustrated in Fig. 4c^33,55^. The GL extract has been blended with PVA/GG (30/70) mixture in 5 wt%. The mechanical properties, water and oxygen permeabilities, water wettability and life cycle of the PVA/GG/GL (S2/GL) bioplastic have been investigated in following sections.

### Chickpea extraction, characterization, and incorporation in PVA/GG bioplastic

Chickpeas are abundant seeds that are grown and consumed all over the world. The seeds are rich in protein and many other nutrients. Protein based bioplastics have been reported as food packaging^29^. Protein-based materials contain extra cross linking that may enhance the bioplastic properties and the hydrophobicity of the material. Ethanolic extract of chickpea seeds has been reported to be rich in phenolic components and oligosaccharides^56,57^. In this paper, we targeted the phenolic and other hydroxy functionalized components in the plants extracts as the -OH functionalized compounds are predicted to act as plasticizers-like materials. Therefore, an aqueous ethanolic solution (50/50) has been used in the extraction of the active components in grounded chickpeas. The FTIR chart for CP aqueous ethanolic extract has been shown in Fig. 4b. The broad peak at 3298 cm^-1^ corresponds to the –OH and –NH stretching vibrations. The peak at 2933 cm^-1^ refers to the –CH and –CH_2_ stretching vibration. The peaks at 1650 and 1565 cm^-1^ are attributed to the amide groups (–CONH) of proteins. The sharp band at 1034 cm^-1^ is referred to the –CH bending of the glycosidic bond^29,58–60^.

The chemical structure of an example of the active compounds present in GL extracts is illustrated in Fig. 4d^61^. The CP extract has been blended with the PVA/GG mixture in 5 wt% and the blend is denoted as S2/CP in this paper. The mechanical properties, water wettability, cytotoxicity, water and oxygen permeability and life cycle of the S2/CP bioplastic have been investigated in following sections.

### Mechanical properties

**Figure 5:**
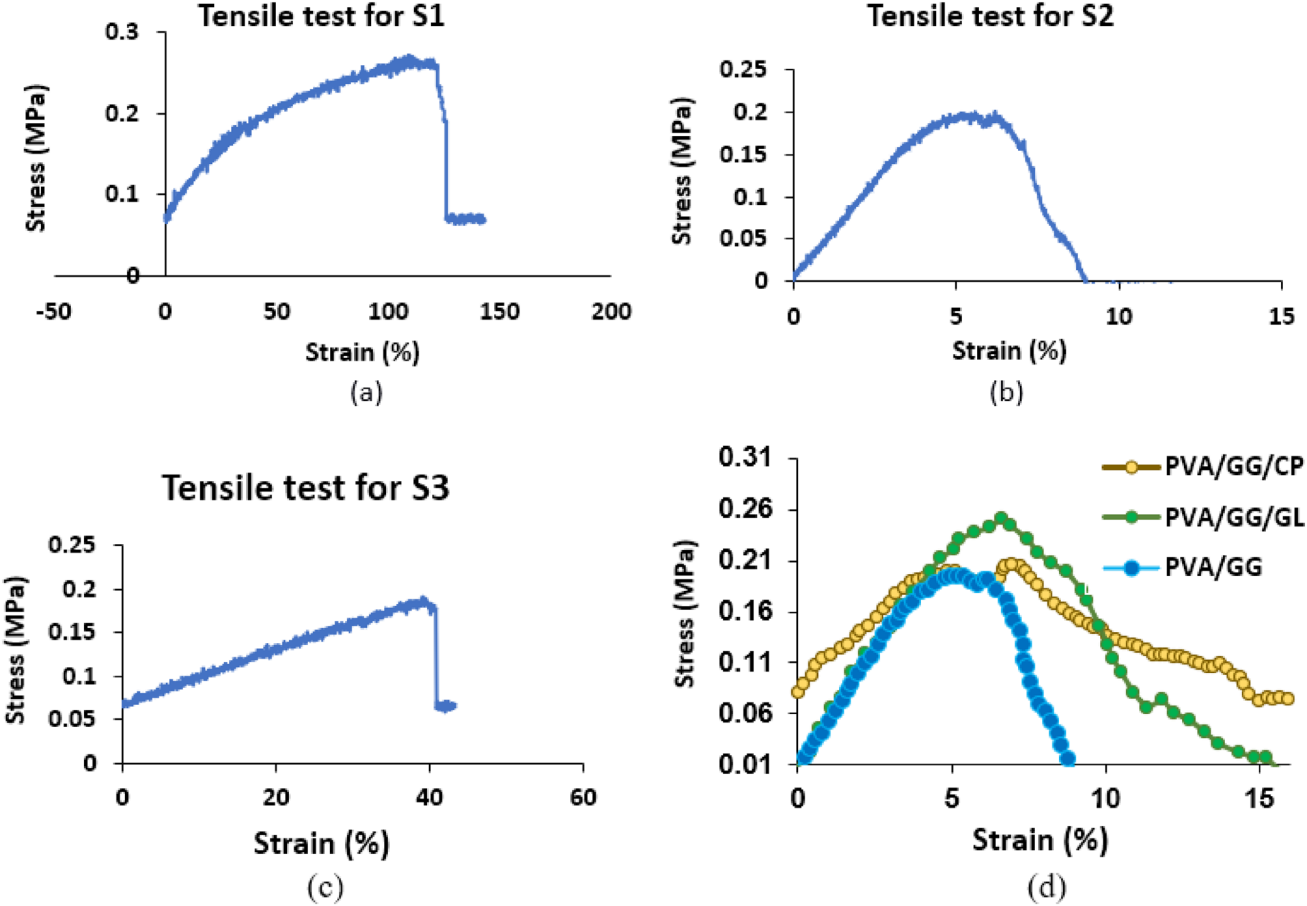
The tensile strength measurement for (**a**) sample S1, (**b**) sample S2, (**c**) sample S3 and (d) S2, S2/CP and S2/GL.

The mechanical properties of the prepared bioplastic have been investigated by measuring the tensile strength for the films S1, S2 and S3 (Table 2) prepared by solvent casting technique. The tensile strength measurements for S1, S2 and S3 have been shown in Fig. 5a–c, respectively. Figure 5d shows the tensile strength for S2, S2/CP and S2/GL.

**Table 2:** The tensile strength and elongation values of the prepared bioplastic films.

Sample (PVA/GG)	TS (MPa)	Strain (%)
S1 (50/50)	0.27	119
S2 (30/70)	0.20	6.19
S3 (70/30)	0.19	39
S2/GL	0.26	6.83
S2/CP	0.22	6.77

From Table 2, S2 had a suitable tensile strength/strain values for a bioplastic material. The sample has the least amount of PVA with a fossil origin, therefore, S2 blend was selected along with S2/plant extracts for further studies. Tensile strength measurements for S2 after addition of the GL and CP extracts have been carried out to investigate the enhancement in the bioplastic mechanical properties by the addition of plant extracts. It has been reported that the addition of PVA to the GG enhanced the GG mechanical properties^16^. After the addition of the GL and CP extracts, the mechanical properties of the bioplastic have been enhanced even further, Fig. 5d. This enhancement can be attributed to the -OH functionalized components in the plant extracts which can act as natural plasticizers. Additionally, the extract components with OH and NH groups can form hydrogen bonds or physical crosslinking with the GG resulting in an improved mechanical property. Similar effects have been observed by other research groups^27,29,62^. The more enhancement in the mechanical properties by addition of GL extract than the CP extract can be attributed to the presence of small molecule phenolic compounds in GL extract.

### Water contact angles measurement

**Figure 6:**
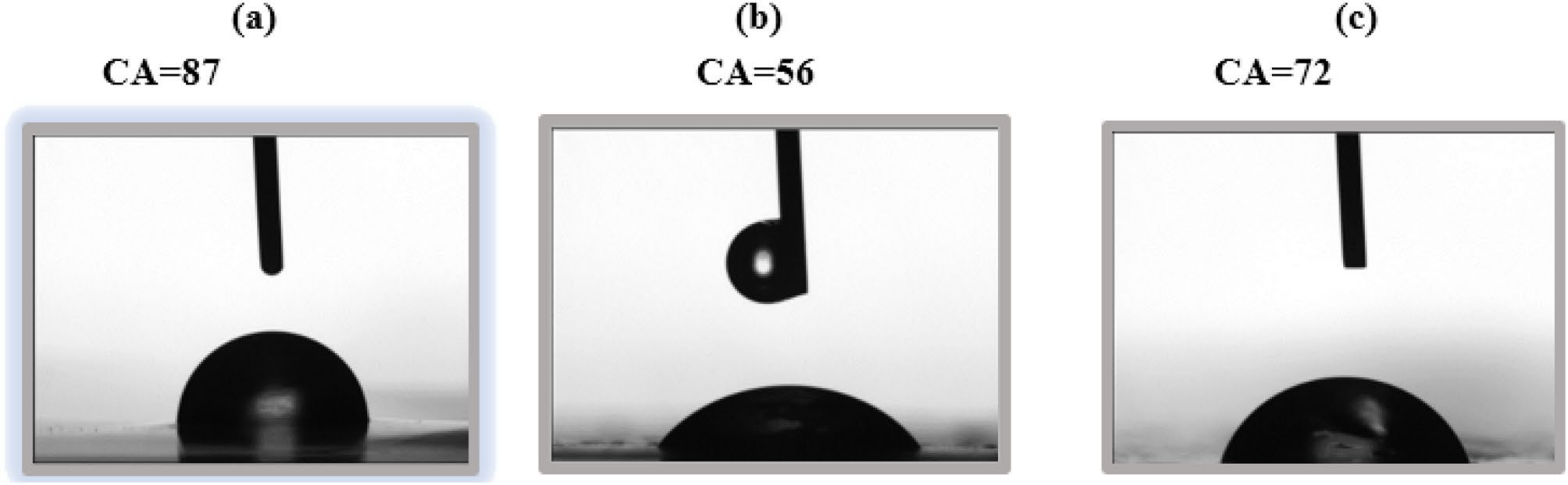
The water contact angle of the bioplastic films: (**a**) S2, (**b**) S2 with GL and (**c**) S2 with CP.

In food packaging, wettability of the plastic plays an important role in the food preservation process and during transportation. A less water wet surfaces are desired in order to keep the packed food safe from moisture^63^. The water contact angle of the PVA/GG bioplastics have been measured, Fig. 6. For the PVA/GG (S2), The contact angle was 87°. The addition of the CP and GL extracts to the PVA/GG blends increased the hydrophilicity of the films. This can be attributed to the more polar components (hydroxyl group functionalities) presented by the addition of the plant extracts. Additionally, film with GL extracts showed more water wetting than that with the CP extracts. This may be attributed to the extraction solvents. GL has been extracted with pure water which may result in the presence of more small polar compounds than those present in CP extract^56,57,63^.

### Cytotoxicity studies

**Figure 7:**
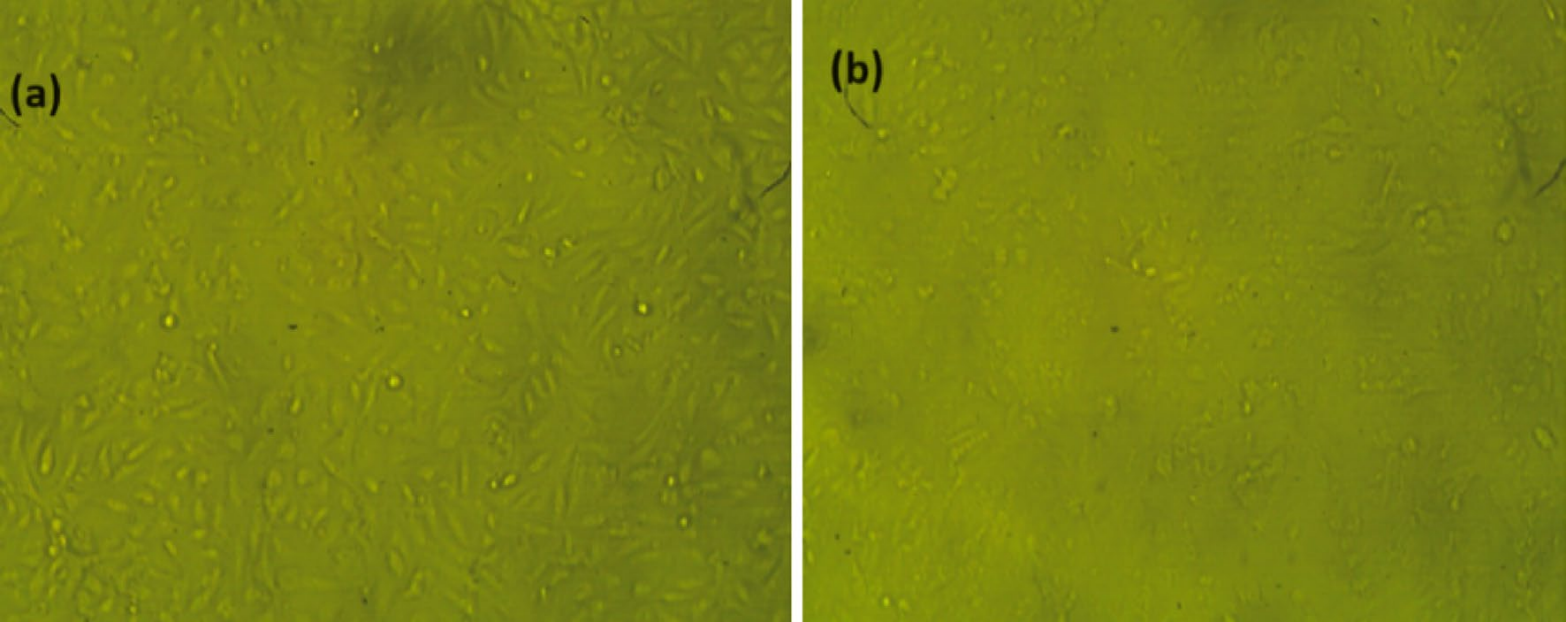
(**a**) Control Vero cells and (**b**) Effect of PVA/GG (S2) on cell viability of Vero normal cell line at concentration 1000 uL and incubation time 24 h.

Cytotoxicity of the film S2 against the Vero normal cell line was assayed. Results showed that the cell viability of Vero cells was 99.67% (Fig. 7 and Table 3), which is considered very safe for S2 to be used in food packaging^63–65^.

**Table 3:** Viability and toxicity of S2 on Vero cell line.

ID	Optical density			Mean optical density	± SE	Viability %*	Toxicity %
Vero	0.717	0.719	0.716	0.716	0.00208	100	0
S2	0.713	0.72	0.7136	0.713	0.00348	99.674115	0.35884

*Viability has been calculated using the formula reported in^42^.

### Scanning electron microscopy (SEM) for the PVA/GG film

**Figure 8:**
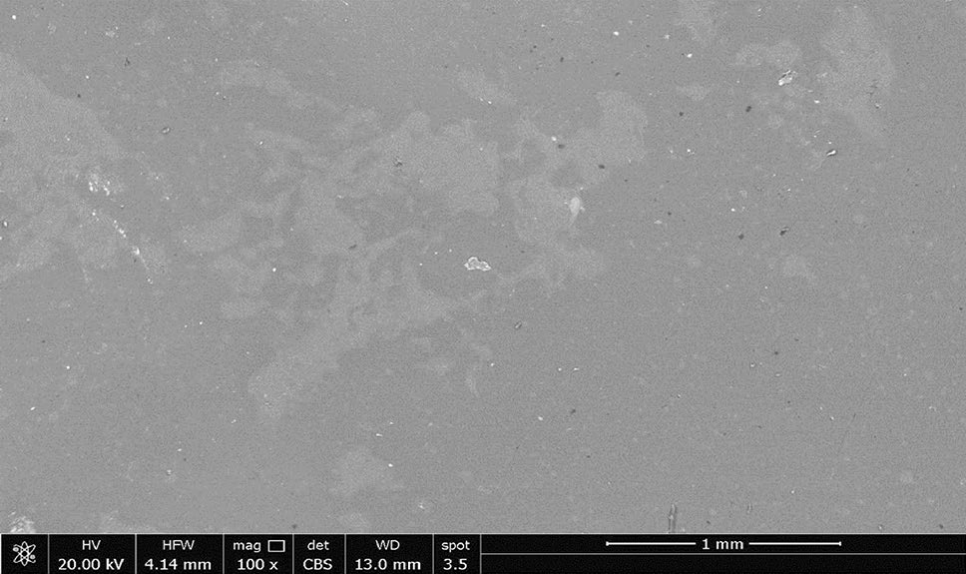
Scanning electron microscopy for S2 bioplastic film.

Scanning electron microscopy for the surface of the sample showed a smooth surface with no cracks, Fig. 8. Only few irregularities or voids have been observed on the surface of the film. The presence of voids might be attributed to the degree of dispersion of the plasticizer which may result in poor crosslinking in few spots of the bioplastic sample^62^. Similar observation in bioplastic films has been reported by Amritkumar et al.^62^ and other research groups^66,67^.

### Water vapor transmission rate (WVTR) measurement

The value of WVTR for the PVA/GG (S2) sample was very comparable to the values of the samples with plant extracts (S2/GL and S2/CP), Table 4. Only slight increase in the WVTR values in the samples with the plant extracts (S2/GL and S2/CP). This might be attributed to the small amount (5 wt%) of the plant extracts added to S2 which caused slight increase in the hydrophilicity of the S2/GL and S2/CP. The plant extracts of GL and CP are rich in hydroxy and phenolic compounds that might cause the slight increase in the hydrophilicity of the samples S2/GL and S2/CP. In general, the bioplastic films, S2, S2/GL and S2/CP exhibited comparable WVTR values to other bioplastics frequently used for food packaging^27,68–71^.

**Table 4:** WVTR values (g/m^2^/d) and oxygen permeability values (g/m^2^/s) for samples S2, S2/GL and S2/CP.

sample	WVTR (g/m^2^/d)	OP (g/m^2^/s)
S2	5.73	18 × 10^−4^
S2/GL	5.77	43 × 10^−4^
S2/CP	5.74	67 × 10^−4^

#### Oxygen permeability (OP)

The oxygen permeability values for samples S2, S2/GL and S2/CP have been listed in Table 4. In general, the OP values noticeably increased in the samples with the plant extracts (S2/GL and S2/CP). It has been reported that the decrease in the hydrogen bonding can lead to a decrease in the oxygen permeability^72^. This observation might explain the noticeable increase in the OP values for S2/GL and S2/CP compared to S2 as the addition of the plants extract might reduce the uniformity of the hydrogen bonding within the samples.

#### Accelerated weathering test

**Figure 9:**
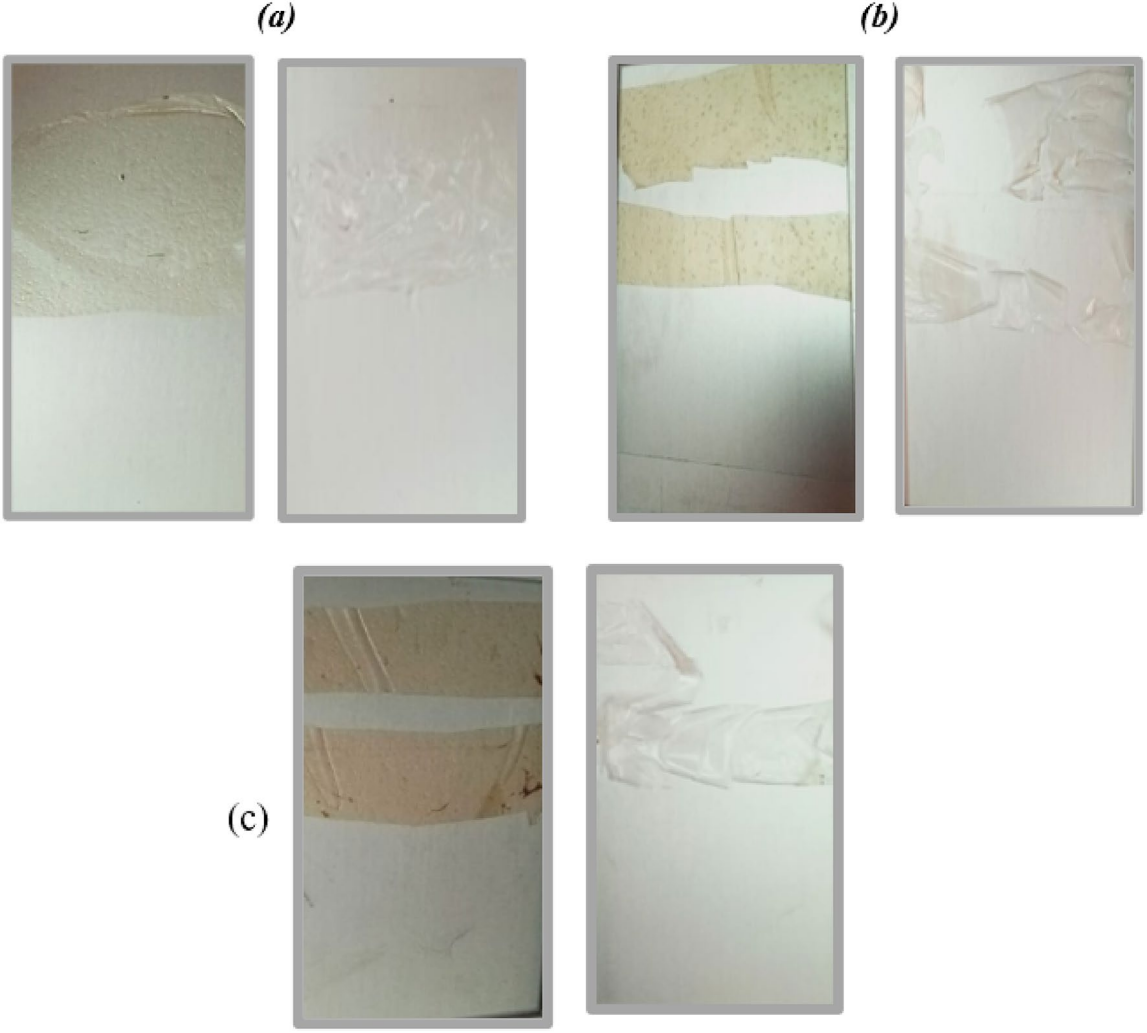
Accelerated weathering test, (**a**) PVA/GG (S2), (**b**) S2 with GL extract and (**c**) S2 with CP extract.

In the accelerated weathering test, it was clear that all the bioplastic samples had undergone a visible change in shape and color, Fig. 9. This indicates the biodegradability of the prepared bioplastics. Additionally, samples with plant extract additives showed more cracks and had been torn. The increased tendency of bioplastics with plant extracts to biodegradation can be attributed to the increased hydrophilic content (ability for hydrolysis) of the samples due to the presence of polar compounds from the plant extracts^73,74^.

#### Life cycle assessment and carbon footprint for the prepared bioplastic

**Figure 10:**
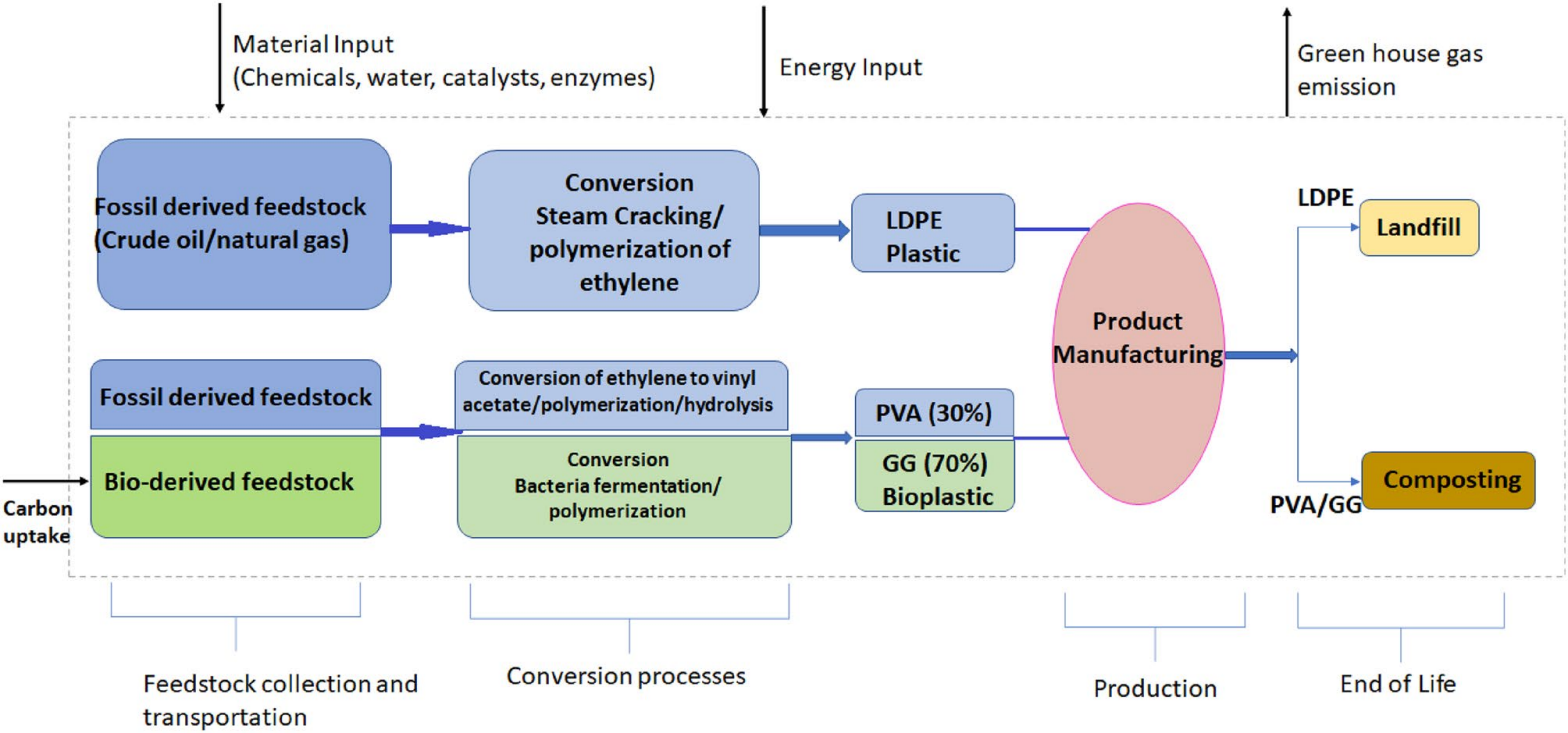
A proposed LCA for the prepared PVA/GG based bioplastic.

Bioplastic is an environmentally friendly solution for the problematic accumulation of fossil-based plastics. The biodegradability of bioplastic is considered an effective solution for plastic waste management. But at the same time, biodegradability led to GHG emissions. Therefore, life cycle assessment (LCA) has become a prerequisite to understand the environmental impact for any newly prepared bioplastics. A proposed LCA for the newly prepared PVA/GG based bioplastic is illustrated in Fig. 10^45,75^. The LCA considered a comparison of a fossil derived plastic, namely LDPE in addition to the newly prepared PVA/GG bioplastics^45^. The PVA/GG based bioplastic composed of 30% PVA and 70% GG, therefore all the assessments and calculations consider the active percentage of each component. The system boundaries in Fig. 10 covered all the materials (chemicals, water, enzymes, etc.) and energy inputs for all stages including the conversion and production process of plastic and possible end of life scenarios. The end-of-life proposed scenarios are landfilling for the LDPE and composting for PVA/GG bioplastic.

To calculate CO_2_ emissions of the bioplastic, detailed GHG for each step and components have been considered and calculated. The GHG values for the bioplastic components and LDPE have been listed in Table 5.

**Table 5:** GHG emissions values for the plastic and bioplastic components.

Component	GHG emission (KgCO_2_e/Kg plastic)	Source of information
PVA	5.5	Ref.^75^
LDPE	2.9	Ref.^45^
GG	7.8	Ref.^76^

**Figure 11:**
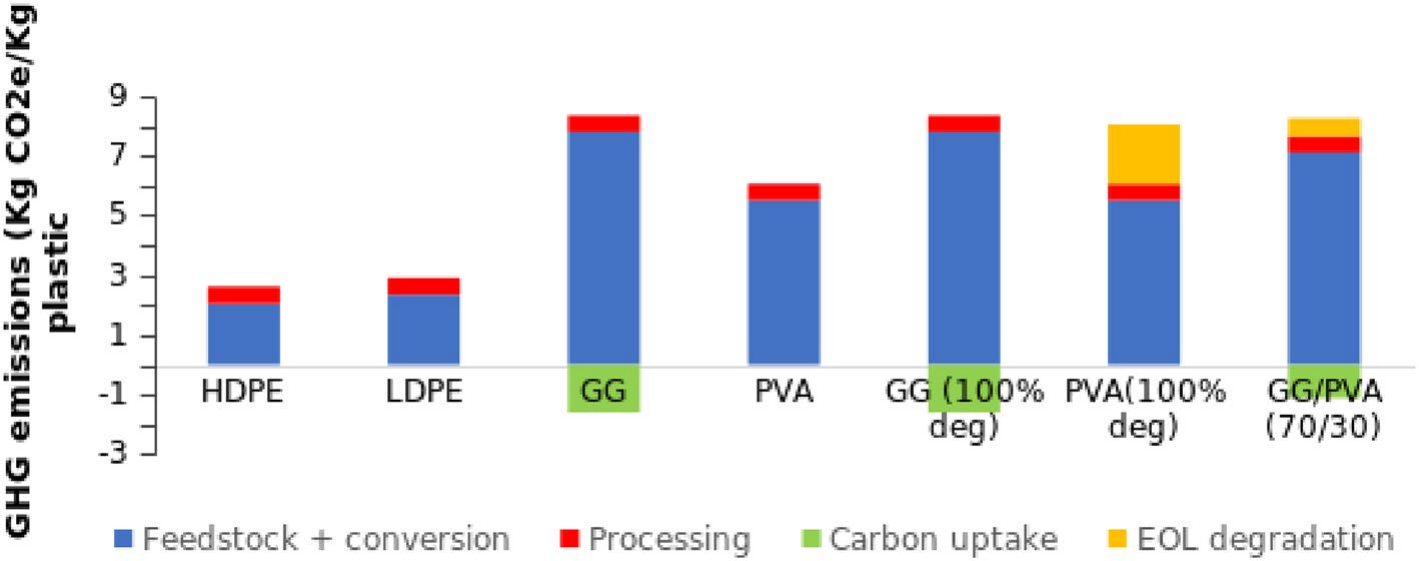
The GHG emissions of PVA/GG based bioplastic in comparison with LDPE and HDPE.

The life cycle GHG emissions of LDPE and the PVA/GG based bioplastic have been illustrated in Fig. 11.

The carbon uptake for GG has been calculated based on the carbon content in its structure (based on its molecular formula) while fossil-based LDPE, HDPE and PVA have no carbon uptake. The net GHG of the bioplastic is then equal to the GHG emissions minus the atmospheric CO_2_ taken or absorbed during the biomass cultivation^45^.

The value of plastic processing and production CO_2_ emission is equal for all plastic and bioplastic as reported by Benavides et al.^45^ after conversion of energy input to CO_2_ emission^77^. The biodegradation of PVA is associated with the emission of 2 kg CO_2_e/Kg PVA^78^. To the best of our knowledge, no exact values for the CO_2_ emissions associated with GG degradation. Consequently, excluding the biodegradation value of GG, the total CO_2_ emissions for the PVA/GG (30/70) bioplastic is equal to 7.14 KgCO_2_e/KG. This value is expected to decrease after the addition of the plant extracts as guava and chickpeas trees are reported to have a potential in increasing carbon sequestration^51^.

## Conclusion

In this work, three compositions of PVA/GG blends have been successfully prepared. The composition with 30/70 percent of PVA/GG showed promising mechanical properties as a bioplastic for food packaging applications. The addition of GL and CP extracts enhanced the mechanical properties even further due to the presence of hydroxy functionalized compounds that can act as natural plasticizers. Scanning electron microscopy studies for the surface of the sample S2 showed normal surface for plastics with few voids that might be attributed to the degree of the dispersion of the plasticizer. Cytotoxicity assay for the new PVA/GG (S2, 30/70) showed that the newly prepared bioplastic is very safe to be used for food packaging. In general, the WVTR test for the S2, S/GL and S2/CP showed values that are comparable to previously reported values for other bioplastics martials for food packaging applications. Oxygen permeability test showed that the addition of plant extracts leads to an increase in the oxygen permeability for samples S2/GL and S2/CP compared to S2 (with PVA and GG only) which might indicate that the compounds present in the added plant extract disturbed the hydrogen bonding between the polymers leading to more gas permeation. The GL and CP bioactive phenolic compounds in their extracts increased the wettability and facilitated the degradation process under the optimum environmental conditions. LCA has been carried out to facilitate carbon footprint calculations. The LCA and carbon footprint calculations revealed that the use biopolymers derived from potential carbon sequestering biomass can largely balance the carbon emissions from bioplastics. Consequently, bioplastic can be a safe solution for fossil based plastic accumulation.

## Data Availability

All data generated or analyzed during this study are included in this article.

## References

[1] Jayasekara R, Harding I, Bowater I, Lonergan G (2005). Biodegradability of a selected range of polymers and polymer blends and standard methods for assessment of biodegradation. J. Polym. Environ..

[2] Song, J., Kay, M., Coles, R. Food and beverage packaging technology, 2nd ed. Chapter 11, Bioplastics, 295–319 (Blackwell Publishing, 2011).

[3] Harzol MD, Sapuan SM, Zainudin ES, Zuhri MYM, Abdul Wahab NI (2021). Corn starch (Zea *mays)* biopolymer plastic reaction in combination with sorbitol and glycerol. Polymers.

[4] Tarique J, Sapuan M, Khalina A (2021). Effect of glycerol plastisizer loading on the physical, mechanical, thermal and barrier properties of arrowroot (maranta arundinacea) starch biopolymers. Sci. Rep..

[5] Birca A, Gherasim O, Grumezescu V, Grumezescu AM (2019). Materials for Biomedical Engineering: Introduction in Thermoplastic and Thermosetting Polymers.

[6] Jariyasakoolroj P, Leelaphiwat P, Harnkarnsujarit N (2020). Advances in research and development of bioplastic for food packaging. J. Sci. Food Agric..

[7] Abdel-Raouf MS, Abdul-Raheim ARM (2017). Removal of heavy metals from industrial wastewater by biomass-based materials: A review. J. Pollut. Eff. Cont..

[8] Hasanin MS, El Saied H, Morsy FA, Rokbaa HHA (2023). Green nanocoating-based polysaccharides decorated with ZnONPs doped Egyptian kaolinite for antimicrobial coating paper. Sci. Rep..

[9] Azmi GE, Saada AM, Shokir EM, El-Deab MS, Attia AM, Omar WEA (2022). Adsorption of xanthan gum polymer and sodium dodecylbenzenesulphonate surfactant in sandstone reservoirs: Experimental and density function theory studies. ACS Omega.

[10] Elsaeed SM, Zaki EG, Omar WAE, Ashraf Soliman AA, Attia AM (2021). Guar gum-based hydrogels as potent green polymers for enhanced oil recovery in high-salinity reservoirs. ACS Omega.

[11] Zia KM, Tabasum S, Khan MF, Akram N, Akhter N, Noreen A, Zuber M (2018). Recent trends on gellan gum blends with natural and synthetic polymers: A review. Int. J. Biol. Macromol..

[12] Vashisth P, Pruthi V (2016). Synthesis and characterization of crosslinked gellan\PVA nanofibers for tissue engineering applications. Mater. Sci. Eng. C.

[13] Suganthi S, Vignesh S, Sundar JK, Raj V (2020). Fabrication of PVA polymer films with improved antibacterial activity by fine-tuning via organic acids for food packaging applications. Appl. Water Sci..

[14] Brunchi C-E, Bercea M, Morariu S, Avadanei M (2016). Investigations on the interactions between xanthan gum and poly(vinyl alcohol) in solid state and aqueous solutions. Eur. Polym. J..

[15] Teixeira MA, Amorim MTP, Felgueiras HP (2020). Poly(vinyl alcohol)-based nanofibrous electrospun scaffolds for tissue engineering applications. Polymers.

[16] Gaaz TS, Sulong AB, Akhtar MN, Kadhum AAH, Mohamed AB, Al-Amiery AA (2015). Properties and applications of polyvinyl alcohol, halloysite nanotubes and their nanocomposites. Molecules.

[17] Tiwari, A., Terada, D., Kobayash, H. Polyvinyl modified guar-gum bioplastics for packaging applications. in *Handbook of Bioplastics and Biocomposites Engineering Applications* 177–188 (Scrivener Publishing LLC, 2011).

[18] Hashem A, El-Naggar ME, Abdelaziz AM, Abdelbary S, Hassan YR, Hasanin MS (2023). Bio-based antimicrobial food packaging films based on hydroxypropyl starch/polyvinyl alcohol loaded with the biosynthesized zinc oxide nanoparticles. Int. J. Biol. Micromol..

[19] Naik ML, Sajjan AM, Ashwini M, Achappa S, Khan TMY, Banapurmath NR, Kalahal PB, Ayachit NH (2022). Nanobacterial cellulose production and its antibacterial activity in biodegradable poly(vinyl alcohol) membranes for food packaging applications. ACS OMEGA.

[20] Rahman H, Mondal IH, Rahman A, Ahmed F, Islam M, Habib A (2022). Recent advancement of PVA/chitosan-based composite biofilm for food packaging. Biomed. J. Sci. Tech. Res..

[21] Imeson A (1997). Thickening and gelling agents for food.

[22] Gong Y, Wang C, Lai RC, Su K, Zhang F, Wang D (2009). An improved injectable polysaccharide hydrogel: Modified gellan gum for long-term cartilage regeneration in vitro. J. Mater. Chem..

[23] Paolicelli P, Petralito S, Varani G, Nardoni M, Pacelli S, Di Muzio L, Tirilli J, Bartuli C, Cesa S, Casadei MA, Adrover A (2018). Effect of glycerol on the physical and mechanical properties of thin gellan gum films for oral drug delivery. Int. J. Pharm..

[24] Safaei HR, Shekouhy M, Rahmanpur S, Shirinfeshan A (2012). Glycerol as a biodegradable and reusable promoting medium for the catalyst-free one-pot three component synthesis of 4H-pyrans. Green Chem..

[25] Epure V, Griffon M, Pollet E, Averous L (2011). Structure and properties of glycerol-plasticized chitosan obtained by mechanical kneading. Carbohydr. Polym..

[26] Sudhamani SR, Prasad MS, Sankar KU (2003). DSC and FTIR studies on gellan and polyvinyl alcohol (PVA) blend Films. Food Hydrocoll..

[27] Tran TN, Mai BT, Setti C, Athanassiou A (2020). Transparent bioplastic derived from CO_2_-based polymer functionalized with oregano waste extract toward active food packaging. ACS Appl. Mater. Interfaces..

[28] Jung H-R, Choi T-R, Han YH, Park Y-L, Park JY, Song H-S (2020). Production pf blue-colored polyhydroxybutyrate (PHB) by one-pot production and coextraction of indigo and phb from recombinant escherichia coli. Dyes Pigments..

[29] Puyana-Perez V, Guartero P, Rosado-Jimenez M, Martinez I, Romero A (2022). Physical crosslinking of pea protein-based bioplastics: Effect of heat and UV treatment. Food Packag. Shelf Life..

[30] Bezerra GF, Rocha JE, Silya MKN, De Freitas TS, De Sousa AK, Dos Santos ATL (2018). Analysis by UPLC-MS-QTOF and antifungal activity of guava (*Psidium Guajava* L.). Food Chem. Toxol..

[31] Shaheena S, Chintaguna AD, Dirisala VR, Kumar NSS (2019). Extraction of bioactive compounds from psidium guajava and their application in dentistry. AMP Expr..

[32] Naseer S, Hussain S, Naeem N, Pervaiz M, Rahman M (2018). The Photochemistry and medicinal value of psidium guajava (guava). Clin. Phytosci..

[33] Kumar NSS, Sarbon NM, Rana SS, Chintagunta AD, Prathibha S, Ingilala SK, Kumar SPJ (2021). Extraction of bioactive compounds from psidium guajava leaves and its utilization in preparation of jellies. AMP Expr..

[34] Luo Y, Liu H, Yang S, Zeng J, Wu Z (2019). Sodium alginate-based green packaging films functionalized by guava leaf extracts and their bioactivities. Materials.

[35] Tassoni A, Tedeschi T, Zurlimi C, Cigognini IM, Petrusan J-I, Rodriguez O (2020). State-of-the art production chains for peas, beans, and chickpeas- valorization of agro-industrial residues and applications of derived extracts. Molecules.

[36] Jayaprakash B, Das A (2018). Extraction and characterization of chickpea (*Cicer arietinum*) extract with immunostimulant acitivity in BALB/C mice. Asian Pac. J. Cancer Prev..

[37] Faridy J-CM, Stephanie C-GM, Gabriels M-MO, Cristian J-M (2020). Biological activities of chickpea in human health (*Cicer arietinum* L.) A review. Plant Foods Hum. Nutr..

[38] Ricci L, Umilta E, Righetti MC, Messina T, Zurlini C, Montanari A, Bronco S, Bertoldo M (2018). On the thermal behaviour of protein isolated from different legumes investigated by DSC and TGA. J. Sci. Food Agric..

[39] Hasanin MS, Youssef AM (2022). Ecofriendly bioactive film doped CuO nanoparticles-based biopolymers and reinforced by enzymatically modified nanocellulose fibers for active packaging applications. Food Packag Shelf Life.

[40] Hasanin M, Abdel Kader AH, Abd El-Sayed ES, Kamel S (2023). Green chitosan-flaxseed gum film loaded with ZnO for packaging applications. Starch.

[41] Joseph TM, Hasanin MS, Unni AB, Mahapatra DK, Haponiuk J, Thomas S (2023). Macromolecules: Contemporary futurist thoughts on progressive journey. Eng.

[42] Hesham AH, Rizk SH, Abdel-Maksoud MA, Al-Qahtani WH, AbdElgawad H, El-Sayyad GS (2023). Unveiling anticancer, antimicrobial, and antioxidant activities of novel synthesized bimetallic boron oxide–zinc oxide nanoparticles. RSC Adv..

[43] Song Z, Xiao H, Zhao Y (2014). Hydrophobic-modified nano-cellulose fiber/PLA biodegradable composites for lowering water vapor transmission rate (WVTR) of paper. Carbohydr. Polym..

[44] Zhang P, Zhao Y, Shi Q (2016). Characterizationof novel edible film based on gum ghatti: Effect of plasticizer type and concentration. Carbohydr. Polym..

[45] Benavides PT, Lee U, Zare-Mehrjerdi O (2020). Life cycle greenhouse gas emissions and energy use of polylactic acid, bio-derived polyethylene, and fossil-derived polyethylene. J. Clean. Prod..

[46] Halim NFA, Majid SR, Arof AK, Kajzar F, Pawlicka A (2012). Gellan gum-lii gel polymer electrolytes. Mol. Cryst. Liq. Cryst..

[47] Zhang N, Li X, Ye J, Yang Y, Huang Y, Zhang X, Xiao M (2020). Effect of gellan gum and xanthan gum synergistic interaction and plasticizers on physical properties of plant based enteric polymer films. Polymers.

[48] Lin S-Y, Cheng W-T, Wei Y-S, Lin H-L (2011). DSC-FTIR microspectroscopy used to investigate the heat-induced intramolecular cyclic anhydride formation between Eudragit E and PVA copolymer. Polym. J..

[49] Mansur HS, Sadahira CM, Souza AN, Mansur AAP (2008). FTIR spectroscopy characterization of poly (vinyl alcohol) hydrogel with different hydrolysis degree and chemically crosslinked with glutaraldehyde. Mater. Sci. Eng. C.

[50] Jamnongkan T, Wattanakornsiri A, Pansila PP, Migliaresi C, Kaewpiron S (2012). Effect of poly(vinyl alcohol)/chitosan ratio on electrospun-nanofiber morphologies. Adv. Mater. Res..

[51] Naik SK, Sarker PK, Das B, Singh AK, Bhatt BP (2021). Biomass production and carbon stock in psidium guajava orchards under hot and sub-humid climate. Current Science.

[52] Shinde SM, Turkhade PD, Deshmukh SB, Narkhede GW (2015). Carbon sequestration potential of some fruit trees in Satara District of Maharashtra India. Econ. Environ. Constr..

[53] Ali LM, Hassan HE, El-Raei AE, Ahmed AA, Saleh SS (2019). The prospect of using guava leaf extract for biosynthesizing chitosan nanoparticles. Adv. Nat. Sci. Nanotechnol..

[54] Somchaidee P, Tedsree K (2018). Green synthesis of high dispersion and narrow size distribution of zero-valent iron nanoperticles using guava leaf (*Psidium Guajava* L.) extract. Adv. Nat. Sci. Nanotechnol..

[55] Simao AA, Marques TR, Marcussi S, Correa AD (2017). Aqueous extract of Psidium guajava leaves: Phenolic compounds and inhibitory potential on digestive enzymes. Ann. Braz. Acad. Sci..

[56] Cheng K, Gao H, Wang R-R, Liu Y, Hou Y-X, Liu X-H, Liu K (2017). Evaluation of extraction and degradation methods to obtain chickpea saponin B1 from chickpea (*Cicer arietinum* L.). Molecules.

[57] Xiaoli X, Liyi Y, Shuang H, Wei L, Yi S, Hao M, Jusong Z, Xiaoxiong Z (2008). Determination of oligosaccharides contents in 19 cultivars of chickpea (*Cicer arietinum* L.) seeds by high performance liquid chromatography. Food Chem..

[58] Sofi SA, Singh J, Muzaffar K, Mir AS, Dar BN (2020). Effect of germination time on physico-chemical functional, pasting, rheology and electrophoretic characteristics of chickpea flour. J. Food Meas. Charact..

[59] Putra RS, Amri RY, Ayu M (2020). Turbidity Removal of Synthetic wastewater Using Biocoagulants Based on Protein and Tannin. AIP Conf. Proc..

[60] Hapani U, Highland HN, Solanki H, George L-B (2021). Extraction of cellulose from lignocellulosic biomass and their application in handmade paper making. Econ. Environ. Constr..

[61] Li P, Shi X, Wei Y, Qin L, Sun W, Xu G, Xu T, Liu T (2015). Synthesis and biological activity of isoflavone derivatives from chickpea as potent anti-diabetic agents. Molecules.

[62] Krishnamurthy A, Amritkumar P (2019). Synthesis and characterization of eco-friendly bioplastic from low-cost plant resources. SN Appl. Sci..

[63] Durmaz BU, Aytac A (2020). Effects of polyol-based plasticizer types and concentration on the properties of polyvinyl alcohol and casein blend films. J. Polym. Environ.ent.

[64] Gorrasi G, Pantani R (2017). Hydrolysis and biodegradation of poly (lactic acid). Polymer.

[65] Maisanaba S, Pichardo S, Jorda-Deneyto M, Aucejo S, Camean AM, Jos A (2014). Cytotoxicity and mutagenicity studies on migration extracts of nanocomposites with potential use in food packaging. Food Chem. Toxicol..

[66] Amin R, Chowdhury MA, Kowser A (2019). Characterization and performance analysis of composite bioplastics synthesized using titanium dioxide nanoparticles with corn starch. Helion.

[67] Lico E, Rapa V, Drushku S, Chatzitheodoridis E (2011). Morphological and chemical study of recycled plastic materials by using scanning electron microscopy and energy dispersive analysis. Mater. Protect.on.

[68] Tran TN, Paul U, Heredia-Guerrero JA, Liakos I, Marras S, Scarpellini A, Ayadi F, Athanassiou A, Bayer IS (2016). Transparent and flexible amorphous cellulose-acrylic hybrids. Chem. Eng. J..

[69] Papadopoulou EL, Paul UC, Tran TN, Suarato G, Ceseracciu L, Marras S, d’Arcy R, Athanassiou A (2019). Sustainable active food packaging from poly (lactic acid) and cocoa bean shells. ACS Appl. Mater. Interfaces.

[70] Tran TN, Heredia-Guerrero JA, Mai BT, Ceseracciu L, Marini L, Athanassiou A, Bayer IS (2017). Bioelastomers based on cocoa shell waste with antioxidant ability. Adv. Sustain. Syst..

[71] Khan MQ, Kharaghani D, Shahzad A, Saito Y, Yamamoto T, Ogasawara H, Kim IS (2019). Fabrication of antibacterial electrospun cellulose acetate/silver-sulfadiazine nanofibers composites for wound dressings applications. Polym. Test..

[72] Poulose S, Jönkkäri I, Hedenqvist MS, Kuusipalo J (2021). Bioplastic films with unusually good oxygen barrier properties based on potato fruit-juice. RSC Adv..

[73] Ortega F, Minnaard J, Arce VB, Garcia MA (2023). Nanocomposite starch films: Cytotoxicity studies and their application as cheese packaging. Food Bioscience.

[74] Siakeng R, Jawaid M, Asim M, Siengchin S (2020). Accelerated weathering and soil burial effect on biodegradability, colour and texture of coir/pineapple leaf fibres/PLA biocomposites. Polymers.

[75] Anthony R, Sharara MA, Runge TM, Anex RP (2017). Life cycle comparison of petroleum- and bio-based paper binder from distillers grains (DG). Ind. Crops Prod..

[76] https://apps.carboncloud.com/climatehub/search?q=gellan%20gum

[77] https://www.epa.gov/energy/greenhouse-gas-equivalencies-calculator#results

[78] https://www.kuraray-poval.com/fileadmin/technical_information/brochures/poval/kuraray-poval-life-cycle-assessment.pdf

